# Flower palate ultrastructure of the carnivorous plant *Genlisea hispidula* Stapf with remarks on the structure and function of the palate in the subgenus *Genlisea* (Lentibulariaceae)

**DOI:** 10.1007/s00709-018-1220-6

**Published:** 2018-02-14

**Authors:** Bartosz J. Płachno, Piotr Świątek, Małgorzata Stpiczyńska, Vitor Fernandes Oliveira Miranda

**Affiliations:** 1 Department of Plant Cytology and Embryology, Jagiellonian University in Kraków, 9 Gronostajowa Str, 30-387 Kraków, Poland; 2 Department of Animal Histology and Embryology, University of Silesia in Katowice, 9 Bankowa Str, 40-007 Katowice, Poland; 3 Botanic Garden, Faculty of Biology, University of Warsaw, Al. Ujazdowskie 4, 00-478 Warsaw, Poland; 4 Faculdade de Ciências Agrárias e Veterinárias, Jaboticabal, Departamento de Biologia Aplicada à Agropecuária, Universidade Estadual Paulista (UNESP), São Paulo, Brazil

**Keywords:** Bladderwort, Carnivorous plant, Corkscrew plants, Floral micro-morphology, *Genlisea*, Glands, Lentibulariaceae, Lamiales, Osmophore, Pollination, Trichome, *Utricularia*, Ultrastructure

## Abstract

In the genus *Genlisea* as well as in its sister genus *Utricularia*, the palate probably plays a key role in providing the colour, mechanical and olfactory stimuli to attract insect pollinators and to guide them to the generative structures and the nectary spur. However, information about the micro-morphology of the palate of *Genlisea* is scarce. This study aims to examine the structure of the palate in *Genlisea hispidula* in detail as well as the palate from other five species from the subgenus *Genlisea*. In particular, its aim is to ascertain whether these palates function as an area for the osmophores in the flower or whether they produce nectar. We showed that the palate in all of the species that were examined was the glandular type and that it had capitate, glandular trichomes, which had a similar general architecture across the species that were examined. No nectar secretion was observed on the palates. The ultrastructure of the palate trichomes showed that the palate glandular trichomes most probably function as scent glands that produce an olfactory stimulus for flower pollinators.

**Electronic supplementary material:**

The online version of this article (10.1007/s00709-018-1220-6) contains supplementary material, which is available to authorized users.

## Introduction

The genus *Genlisea* A.St.-Hil. includes approximately 30–31 species that are classified into two subgenera—*Genlisea* and *Tayloria*. *Genlisea* are small herbaceous rootless plants that form specific traps to catch small soil/water organisms (e.g. Reut 1993; Płachno et al. 2007; Fleischmann 2012a; Fleischmann et al. 2011, 2017, and references therein).

**Fig. 1:**
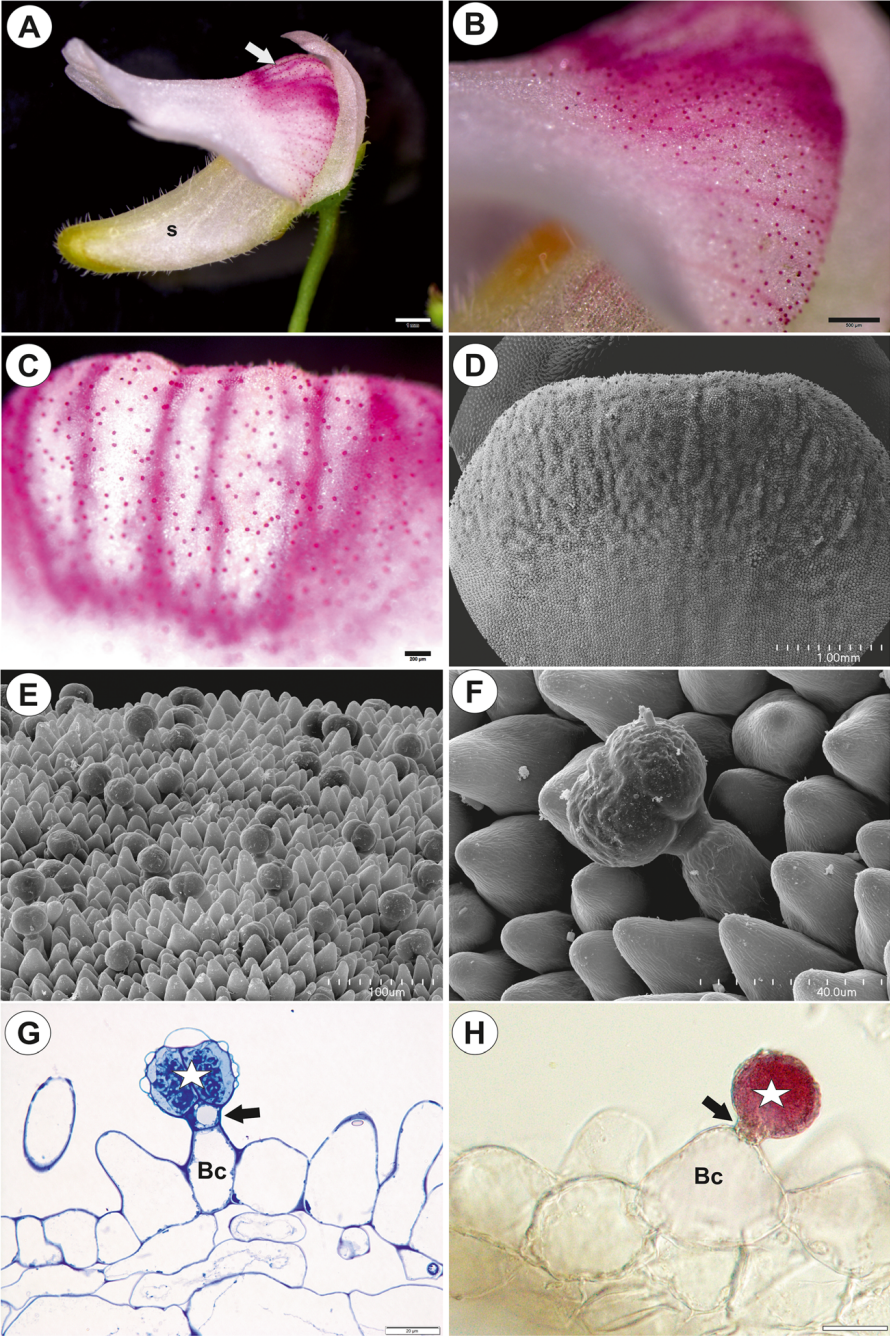
General floral morphology and palate micromorphology of *Genlisea hispidula*. **a** General floral morphology of *G. hispidula*, palate (arrow), spur (s); bar = 1 mm. **b**, **c** Micromorphology of the palate, note the numerous glandular trichomes; bar = 500 and 200 μm. **d** Micromorphology of the palate in SEM; bar = 1 mm. **e**, **f** A section of the palate with glandular trichomes and papillae; bar = 100 and 40 μm. **g** Part of the section through the palate with a glandular trichome and papillae, note the subcuticular spaces of the head cells: pedestal cell (arrow), basal cell (Bc), head (star); light microscopy (LM). Semi-thin section, stained with methylene blue, bar = 20 μm. **h** Part of the section through the fresh palate showing accumulations of anthocyanins in the head cells of the trichome: pedestal cell (arrow), basal cell (Bc), head (star); bar = 20 μm

*Genlisea* have zygomorphic flowers in which the chasmogamous and bilabiate corolla is made up of five fused petals with a spur. The upper lip is created by the fusion of two petals and the lower lip by the fusion of three petals. The lower lip is flat, extended and forms a palate at the base and later a throat that hides the sexual organs and extends to the spur (Fromm-Trinta 1979; Fleischmann et al. 2011; Fleischmann 2012a). According to Fleischmann (2012a, 2012b), the species of *G.* subgenus *Genlisea* are characterised by masked flowers—“snap-dragon blossoms”. The palate forms a gibbous mask, which is firmly appressed to the upper lip and blocks access to the entrance to the throat (corolla tube) and nectar spur (Fig. 1a–c). There are only rare observations of the pollinators of the *G*. subgenus *Genlisea*; small bees (family Halictidae) are the pollinators of the African *G. stapfii* and larger bees (family Megachilidae) are visitors to the Brazilian *G. aurea* (Fleischmann 2012, 2012a).

The species of the *G.* subgenus *Tayloria* have a different corolla design than the one in the subgenus *Genlisea* (a flower with a long slender corolla tube with a narrowed entrance and disc-like spreading corolla lobes) (Fleischmann 2012b). Fleischmann (2012a, 2012b) suggested that the flowers of the members of the subgenus *Tayloria* should be pollinated by insects with a long proboscis such as butterflies, moths or by dipterans such as bombyliid flies. However, Aranguren et al. (2017) found that *G. violacea* flowers were mainly pollinated by the bees *Lasioglossum* sp. (Halictidae) and *Ceratina* sp. (Apidae) and also the flies *Toxomerus* (Syrphidae). These flowers were also visited by some species of Diptera, Lepidoptera and Hymenoptera.

In *Utricularia* (the genus most related to *Genlisea*), there are large differences between the species of pollinators. For example, species that have small flowers such as *U. albocaerulea*, *U. purpurascens* and *U. reticulata* (Hobbhahn et al. 2006) were visited by various insects: bees, butterflies, moths and flies, and some species of Hymenoptera, which were effective pollinators. In *Utricularia bremii*, small Hymenoptera (Mymaridae, Braconidae) were observed as flower visitors (Płachno et al. 2017a). The large flowered *Utricularia* species, e.g. *U. reniformis* (Clivati et al. 2014), *U. cornigera* and *U. nelumbifolia* (Płachno et al. 2017b), have large, strong pollinators that have easy access to their sexual organs and nectar. Clivati et al. (2014) observed large bees (the carpenter bee *Xylocopa* sp. and the bumblebee *Bombus* sp.) as pollinators of *U. reniformis* flowers.

In *Utricularia* flowers, the palates are morphologically very diverse (Taylor 1989) and probably play a key role in providing the colour, mechanical and olfactory stimuli to attract insect pollinators and to guide them to their generative structures and the spur with nectar (Płachno et al. 2016, 2017a,b). The palates in *Genlisea* also provide the colour signals for pollinators (Fleischmann 2012); however, information about the micro-morphology of *Genlisea* palates is scarce. Fleischmann (2012a) observed glandular trichomes on the palate surface of *Genlisea subglabra* and interpreted them as nectar glands.

This study aims to examine the structure of the palate in *Genlisea hispidula* in detail as well as the palate from other five species from the subgenus *Genlisea*. In particular, it aims to ascertain whether these palates function as a unguentarius (Płachno et al. 2017a; = area of osmophores in the flower; Endress 1994) or whether they produce nectar to attract flower visitors. It should be stressed that is interesting to compare the glandular trichomes from the *Genlisea* palate to the *Utricularia* palate due to their close systematic relationship.

## Material and methods

The species that were used in this study include *G. hispidula* Stapf, *G. subglabra* Stapf, *G. roraimensis* N.E.Br. and *G. africana* Oliv, which were obtained from the collections of Botanická zahrada hl. m. Prahy, Czech Republic; Botanická zahrada Liberec, Czech Republic, the collection of Mr. Kamil Pasek (bestcarnivorousplants.com); and the Botanical Garden of Jagiellonian University in Kraków, Poland. The *Genlisea repens* Benj. and *G. pygmaea* A.St.-Hil. Material was collected by BJP and VFOM from populations that are located in the southern region in the rocky fields of the cerrado of Minas Gerais State in the Serra da Canastra (south-eastern Brazil), permit no. SISBI0#26938. Flowers of these species were fixed in the field condition.

### Floral structure and histochemistry

The distribution of the secretory glandular trichomes was determined by examining whole flowers using an Olympus SZX16 stereoscopic microscope (equipped with an Olympus DP72 camera and the cell Sens Standard 1.4 program). Floral parts that bore glandular trichomes, namely the palate of *G. hispidula*, were examined using light microscopy (LM), scanning electron microscopy (SEM) and transmission electron microscopy (TEM) as follows. First, the epidermis of the floral palate was examined during anthesis and pieces of the floral tissues were excised and fixed in 2.5% glutaraldehyde and 2.5% formaldehyde in a 0.05 M cacodylate buffer (Sigma) (pH 7.2) overnight (material from cultivated plants) or for several days (material from plants collected in the field condition), washed three times in a 0.1 M sodium cacodylate buffer and post-fixed in a 1% osmium tetroxide solution for 1.5 h at room temperature. This was followed by dehydration using a graded ethanol series and infiltration and embedding using an epoxy embedding medium kit (Fluka). Following polymerisation at 60 °C, sections were cut at 70 nm for TEM using a Leica ultracut UCT ultramicrotome, stained with uranyl acetate and lead citrate (Reynolds 1963) and examined using a Hitachi H500 transmission electron microscope at an accelerating voltage of 75 kV.

Semi-thin sections (0.9–1.0 μm thick) were prepared for LM and stained for general histology using aqueous methylene blue/azure II (MB/AII) for 1–2 min (Humphrey and Pittman 1974) and examined with an Olympus BX60 light microscope. The periodic acid-Schiff (PAS) reaction was also used to reveal the presence of insoluble polysaccharides, and Sudan Black B was used to detect the presence of lipids (Jensen 1962). Staining for total proteins was performed using Coomassie brilliant blue R250 or Ponceau 2R (Fisher 1968; Ruzin 1999).

Nikon Eclipse Ni-U and Olympus BX60 microscopes were used for the general photography and micrometry/photomicrography, respectively.

For SEM, the representative floral parts of six *Genlisea* species were fixed (as above) and later dehydrated and subjected to critical-point drying using liquid CO_2_. They were then sputter-coated with gold and examined at an accelerating voltage of 20 kV using a Hitachi S-4700 scanning electron microscope (Hitachi, Tokyo, Japan), which is housed at the Institute of Geological Sciences, Jagiellonian University in Kraków.

## Results

### *G. hispidula* (Figs. 1, 2 and 3)

The palate was prominent with clearly visible trichomes on the adaxial surface (Fig. 1a–d). Although the palate epidermis formed papillae (Fig. 1d, e), they were not on the entire surface of the palate. There were capitate, glandular trichomes between the papillae (Fig. 1e–g) (length about = 70 μm) consisting of a unicellular basal cell, a very short, pedestal cell (= barrier cell) (length about = 11 μm) and a head comprising two glandular cells (diameter of the head about = 40 μm) (Fig. 1g, h). The lateral wall of the basal cell was partly embedded in the epidermis, but most of this cell protruded to form a long stalk, which had a thick cuticle. However, in some of the trichomes, the basal cell had a more trapezoid shape (Fig. 1h). The basal cell was highly vacuolated, and the vacuole was translucent or contained pink anthocyanins. The cytoplasm in the basal cell formed a thin layer that contained the usual organelles.

**Fig. 2:**
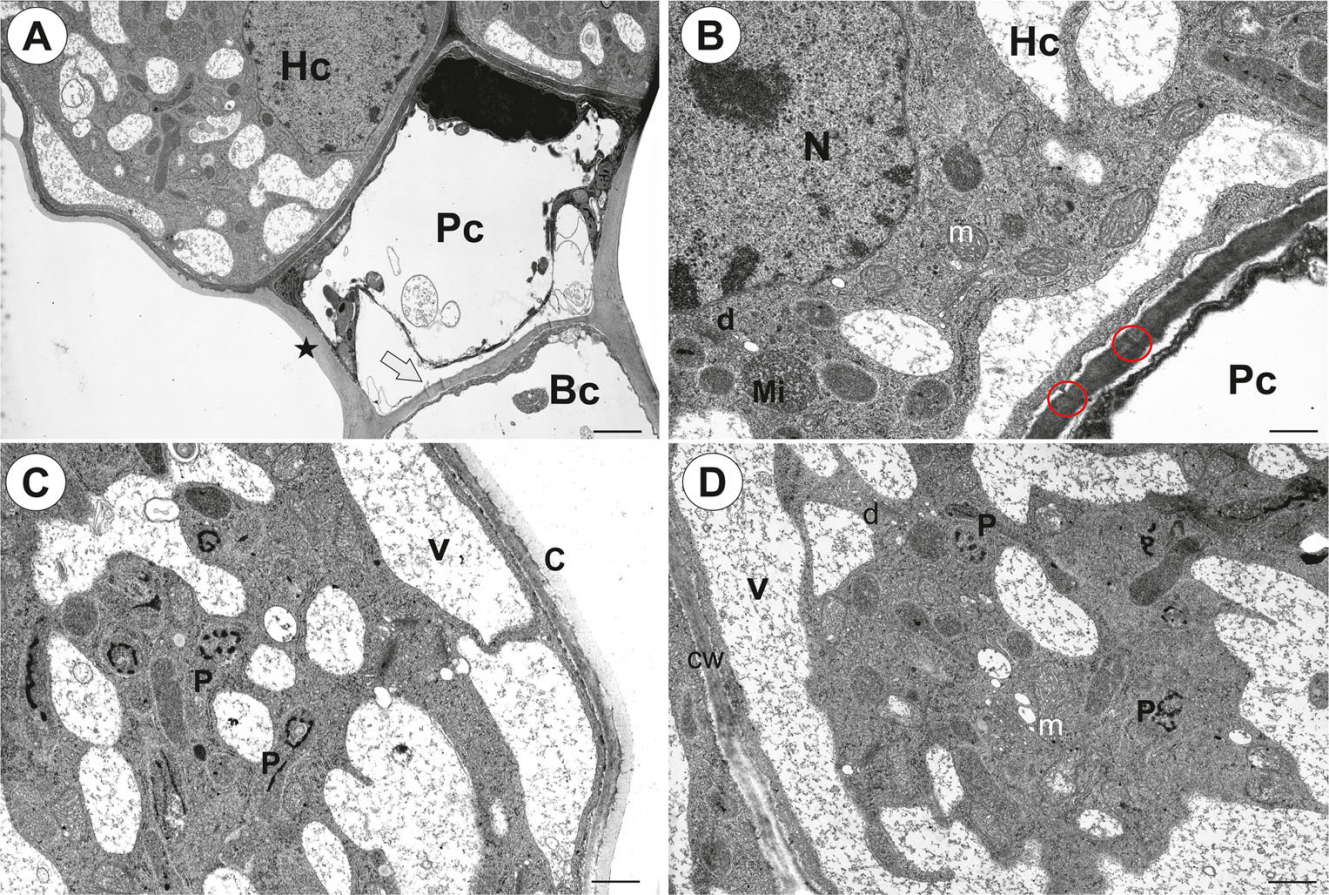
Ultrastructure of the palate trichomes of *Genlisea hispidula* from an immature flower. **a** Longitudinal section showing the head cells (Hc), pedestal cell (Pc), basal cell (Bc), thickened anticlinal wall of stalk cell (star); bar = 2 μm. **b** Ultrastructure of the pedestal (Pc) and head cell (Hc): nucleus (N), microbody (Mi), dictyosome (d), plasmodesmata (encircled); bar = 0.75 μm. **c**, **d** Ultrastructure of the head cells: plastids (P), vacuole (V), mitochondria (m), cell walls between the terminal cells (cw), cuticle (c); bar = 0.75 and 1 μm

**Fig. 3:**
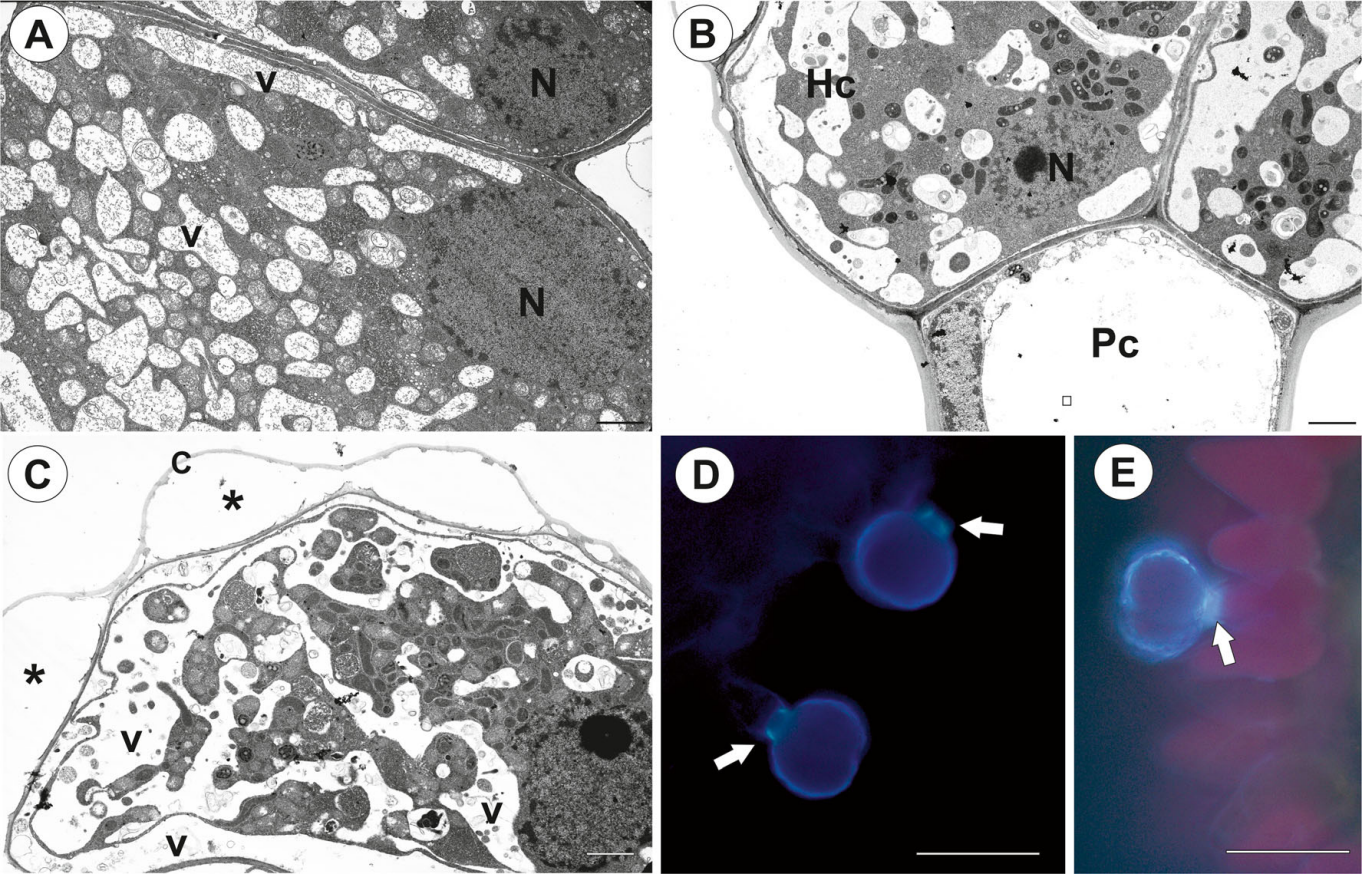
Structure of the palate trichomes of *Genlisea hispidula*. **a** Longitudinal section showing the head cells of trichome from an immature flower: nucleus (N), vacuole (V); bar = 2 μm. **b** Ultrastructure of the pedestal (Pc) and head cells (Hc) of the trichome from a mature flower: nucleus (N); bar = 1.7 μm. C. Ultrastructure of the head cell of the trichome from a mature flower: cuticle (c), subcuticular space (asterisk), vacuole (V); bar = 1.7 μm. **d**, **e** The strong auto-fluorescence of the cuticles of the head cells under UV; florescence microscopy, bars = 25 μm

The pedestal cell had a thick radial wall in which cutinisation occurred that led to its transformation into Casparian strip-like structures (Fig. 2a). These cuticular deposits of the pedestal cell were continuous with a well-developed cuticle of the glandular cells (Fig. 2a). Simple plasmodesmata occurred in the transverse walls between the basal cell and the pedestal cell (Fig. 2a). There were branched plasmodesmata in the transverse walls between the pedestal cell and the head cells.

There were two glandular head cells. The cytoplasm of the head cells was mostly concentrated towards its base and radial walls (Figs. 2a and 3a, b). A prominent nucleus was localised here. There were numerous mitochondria, microbodies and small dictyosomes in the cytoplasm (Fig. 2b–d). There were numerous plastids that contained small plastoglobuli and lamellae with osmiophilic inclusions. Although small lipid droplets were visible in the cytoplasm of the head cells, they were not frequent (not shown). The most conspicuous character of the head cell was a reticulate vacuolar system (Figs. 2a–d and 3a–c). Analysis of ultra-thin sections suggested that it was divided into various small but interconnected parts (e.g. Fig. 3d). Vacuolisation increased during flower development (immature flower Fig. 2a; mature flower Fig. 3b, c). The cuticle of the head cell was thick (mean thickness = 0.225 μm; Figs. 2c and 3c) and contained microchannels. The subcuticular spaces were formed by the separation of the cuticle from the cell wall (Fig. 3c). The folded cuticle was also clearly visible in SEM (Fig. 1f), as well was clearly visible in the living trichomes (Fig. 1h). The cuticles of the head cells emitted a strong auto-fluorescence under UV (Fig. 3d, e), and this might reflect differential impregnation of cell walls with cutin and other materials between glandular cells and other cell types. Cytochemical tests did not show that the cell head produced insoluble polysaccharides or protein products (Sup. Material 1). Treatment for lipids stained the plastids and cuticle of head cells and lateral wall of pedestal cell (not shown).

### Other species (Fig. 4a–f)

**Fig. 4:**
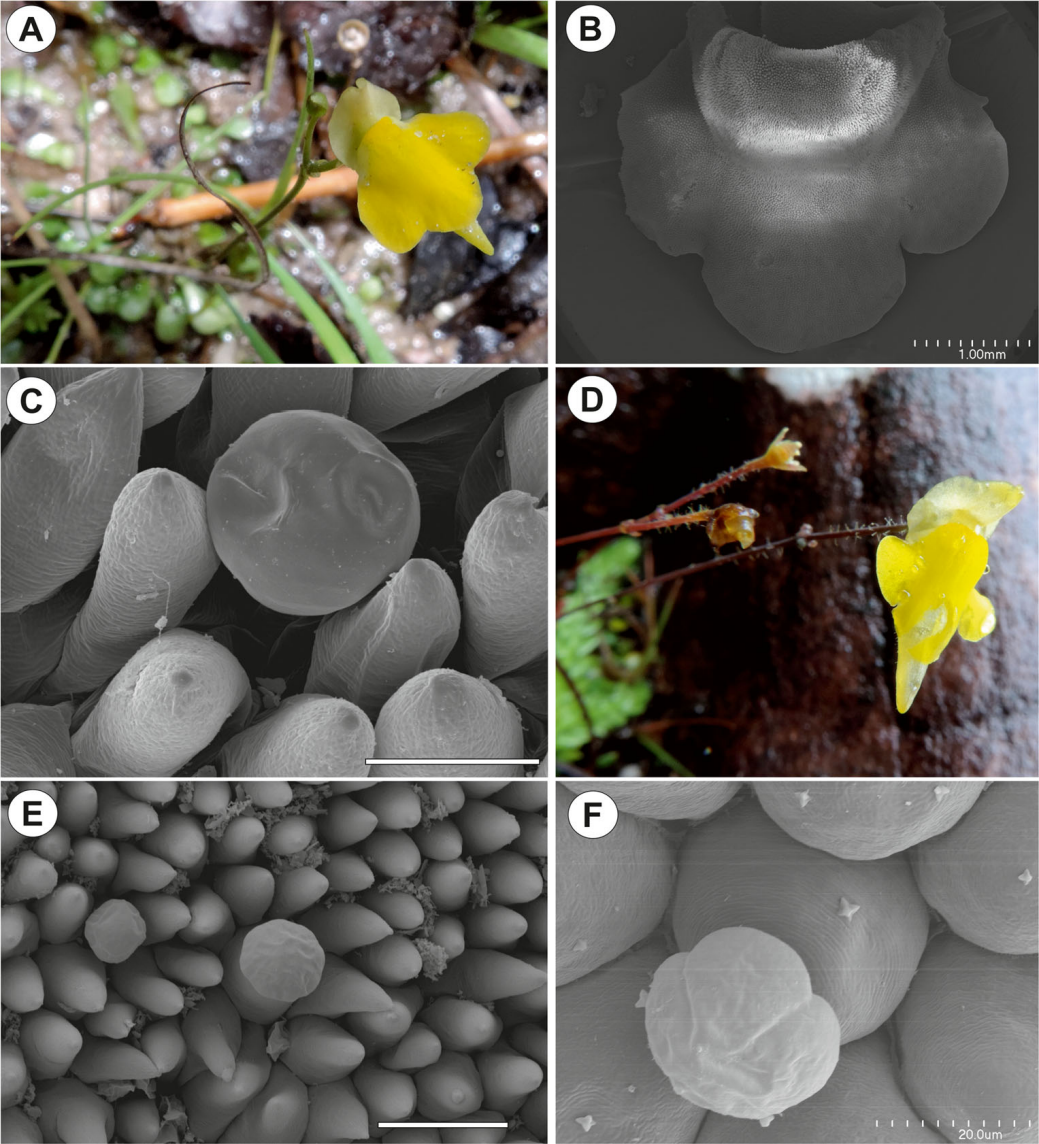
Morphology of *Genlisea* flowers. **a** Floral morphology of *Genlisea repens* in the southern region of Minas Gerais State in the Serra da Canastra. **b** Morphology of the lower lip of a *G. repens* flower; bar = 1 mm. **c** A section of the *G. repens* palate with a glandular trichome and papillae; bar = 20 μm. **d** Floral morphology of *Genlisea pygmaea* in the southern region of Minas Gerais State in the Serra da Canastra. **e** A section of the *G. pygmaea* palate with a glandular trichome and papillae; bar = 50 μm. **f** Glandular trichome from palate of *G. africana*; bar = 20 μm

In the palates of all of the other species (*G. subglabra*, *G. roraimensis*, *G. africana*, *G. repens* (Fig. 4a) and *G pygmaea* (Fig. 4d)) that were examined, the adaxial epidermal surface consisted of conical papillae (Fig. 4b, c, e). There were capitate, glandular trichomes between the papillae (Fig. 4c, e, f). Each trichome consisted of a basal cell, a pedestal cell and a multi-celled head. Compared to *G. hispidula*, the other species that were examined had a more rounded head shape (Fig. 4c, e, f).

## Discussion

Fleischmann (2012a) interpreted the glandular trichomes on palate surface of *G. subglabra* as nectar glands. However, in vivo no nectar was observed on these trichomes in this species or on the palate trichomes of the other species that were examined in this study. Also, PAS reaction results did not reveal that these hairs produce polysaccharide secretion. We also did not find any proof to confirm Fleischmann’s suggestion. These trichomes did not produce polysaccharides or protein products. The micro-morphology of the *Genlisea violacea* palate was analysed recently (Aranguren et al. 2018). However, this species from the subgenus *Tayloria* did not have capitate trichomes on its palate. Future studies of other species of the subgenus *Tayloria* should reveal whether there is any structural pattern of palate morphology in this subgenus. We should mention that here, we examined in details only *G. hispidula* trichomes (TEM data); thus, other species should be studied in case of ultrastructure in the future. This could increase value of the comparative analysis.

Stalked glandular trichomes with a bicellular head, which are similar to the palate trichomes of *G. hispidula* and *G. subglabra*, were described from the palate of *Utricularia cornigera* and *U. nelumbifolia* (Płachno et al. 2017b). However, these trichomes did not have a folded cuticle of the head cells, which was observed in the *Genlisea* trichomes. In our previous studies, we described the ultrastructure of the short-stalked or sessile glandular trichomes from an area of the osmophores (unguentarius) in *Utricularia dunlopii* (Płachno et al. 2016), *U. bremii* and *U. minor* (Płachno et al. 2017a). In some characters, these trichomes are similar to the palate trichomes of *Genlisea*. This might suggest that they have a similar function. For example, plastids that have lipid globules of the head cells of *G. hispidula* were similar to those that were found in *U. dunlopii* and *U. bremii*. Moreover, in the trichomes of these three species, TEM observations revealed that the cuticle became distended and separated from the cell walls of the head cells and formed a subcuticular space. A leucoplast that contained lamellae with osmiophilic inclusions was described from the secretory cell of the trichomes that produce phenols and terpenoids in *Tussilago farfara* and other Asteraceae species (Muravnik et al. 2016, and literature therein). Muravnik et al. (2016) suggested that the black deposition in the lamellae correspond to terpene precursors.

In the head cells of *Genlisea*, the trichomes were numerous microbodies. It is believed that microbodies actively participate in the production of secondary metabolites such as terpenoids and phenolic substances (e.g. Vassilyev 2000; Muravnik et al. 2016). The lipid bodies in the cytoplasm and/or plastids with lipid globules were frequently recorded in osmophore tissues (e.g. Pridgeon and Stern 1983; Stern et al. 1987; Vogel 1990; Ascensao et al. 2005; Płachno et al. 2010; Antoń et al. 2012).

Although the tissue of the *Utricularia dunlopii* unguentarius was rich in starch grains, which was similar to the starch that was recorded in the parenchyma cells of the *U. bremii* palate, this is in contrast to the *G. hispidula* palate, in which we did not observed any starch. In the glandular trichomes from the area of the osmophores from *U. dunlopii* (Płachno et al. 2016) and the palate of *U. bremii* (Płachno et al. 2017a), both the barrier cell and head cells were transfer cells (the occurrence of cell wall ingrowths). In the *G. hispidula* palate trichomes, we did not find any transfer cells. However, cell wall ingrowths were recorded in the cells of the trichomes that occur in *Genlisea* traps (Heslop-Harrison 1976; Płachno et al. 2007).

The most unusual character of the head cells of the palate trichomes in *G. hispidula* was the specific vacuolisation. Although a vacuole with some cytoplasmic bridges was observed in the head cells of *Utricularia dunlopii* trichomes (see Fig. 3a in Płachno et al. 2016). Observations of ultra-thin sections suggested that the vacuole in *Genlisea* was extremely divided into various small but interconnected parts (e.g. Fig. 3c). However, the analysis of series of TEM sections and preparation of 3D reconstruction have to be done to proof whether these small vacuoles are really interconnected. Such a reticulate vacuole may be a character of cell development, e.g. in the cells of the barley root meristem (Guilliermond 1941) or it may be a symptom of special cell activity. Lazzaro and Thomson (1996) described a complicated vacuolar-tubular system in the cells of *Cicer arietinum* trichomes. These authors proposed that the vacuolar-tubular system in these trichomes functions to rapidly deliver solute from the base of the trichome to the secretory head cells. A specific process called aggregation was described in carnivorous plant glands (Juniper et al. 1989; Peroutka et al. 2008, and literature therein). After the chemical and mechanical stimulation of the glands, a large central vacuole was cleaved into various small but interconnected parts by the cytoplasm in the gland cells. Although there are various hypotheses about the function of this process, these changes provide evidence of a high level of activity of these cells. Thus, we also believe that the occurrence of a complex vacuolar system in the head cells of *Genlisea* trichomes is connected with a high level of metabolic activity (production and secretion).

## Conclusion

Based on our morphological and ultrastructural observations, we suggest that the palate in *Genlisea* from the subgenus *Genlisea* may function as an area of osmophores in the flower that provide an olfactory stimulus for pollinators.

## Electronic supplementary material

Supplementary Material 1 A section of the *G. hispidula* palate after PAS reaction. The PAS reaction did not indicate that the cells of trichome head produced insoluble polysaccharide secretion; bar = 20 μm. (GIF 8587 kb)

High resolution image (TIFF 15076 kb)
