# Supplementary figures and images for: Flower palate ultrastructure of the carnivorous plant Genlisea hispidula Stapf with remarks on the structure and function of the palate in the subgenus Genlisea (Lentibulariaceae)
Source: Protoplasma. 2018 Feb 14;255(4):1139–46. doi: 10.1007/s00709-018-1220-6 (PMC5994213; doi:10.1007/s00709-018-1220-6)

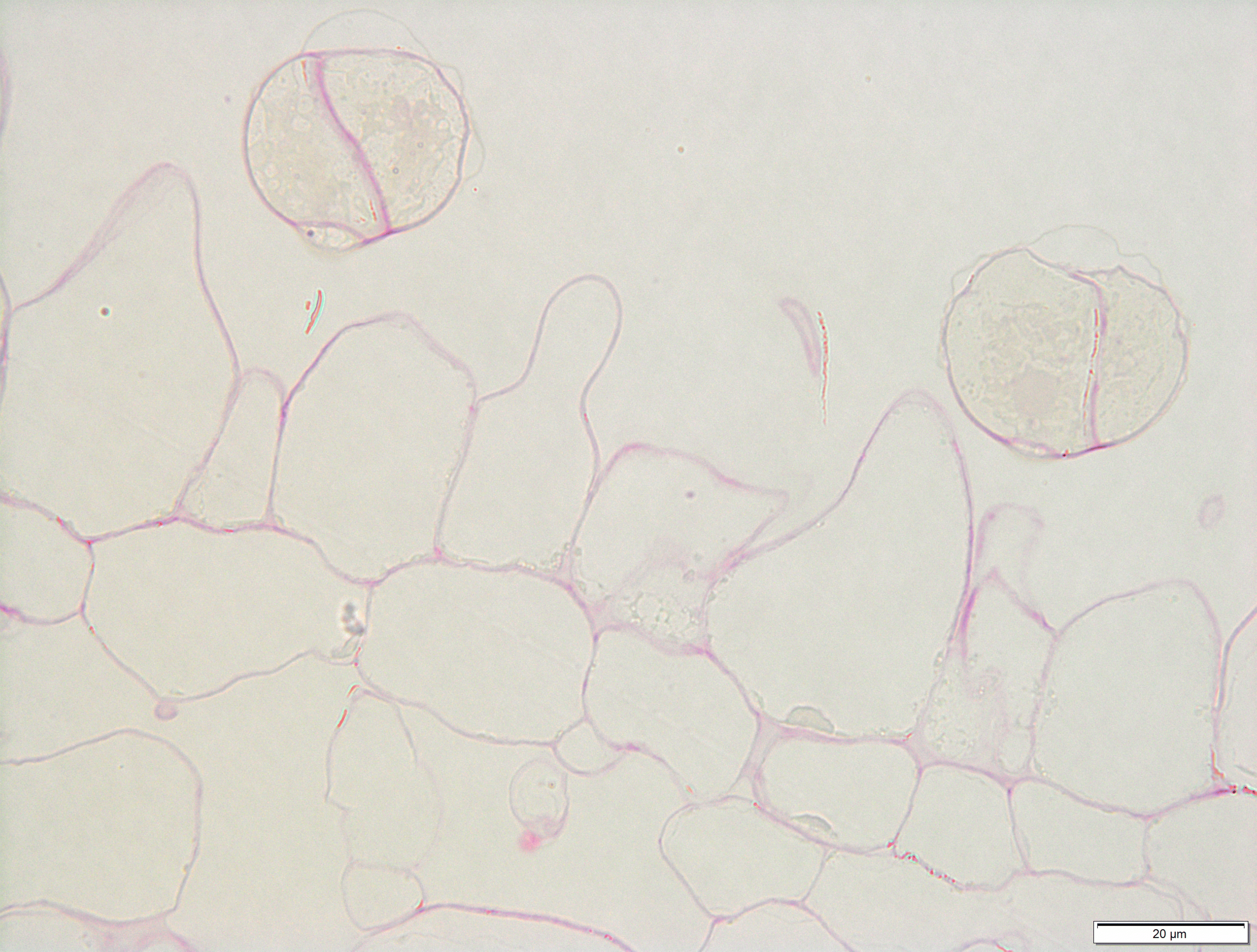

Supplement: Supplementary file 1 — A section of the G. hispidula palate after PAS reaction. The PAS reaction did not indicate that the cells of trichome head produced insoluble polysaccharide secretion; bar = 20 μm. (GIF 8587 kb) [file 709_2018_1220_Fig5_ESM.gif]

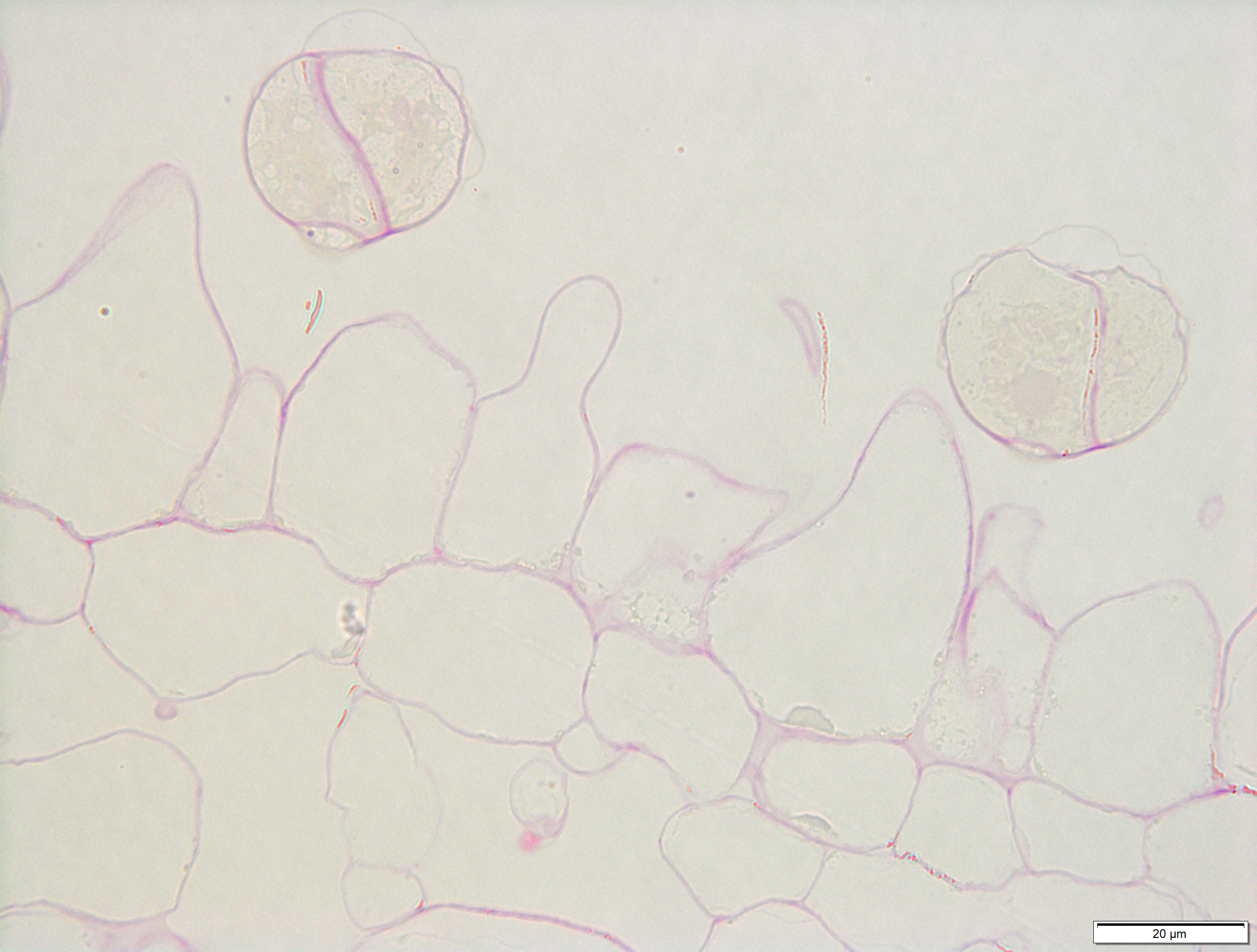

Supplement: Supplementary file 2 — High resolution image (TIFF 15076 kb) [file 709_2018_1220_MOESM1_ESM.tif]
